# Prediction of In-Hospital Mortality in Patients With Traumatic Brain Injury Using the Rotterdam and Marshall CT Scores: A Retrospective Study From Western India

**DOI:** 10.7759/cureus.41548

**Published:** 2023-07-08

**Authors:** Brijesh Goswami, Vivek Nanda, Sharvilkumar Kataria, Deeti Kataria

**Affiliations:** 1 Department of Emergency Medicine, Apex Emergency Hospital, Ahmedabad, IND; 2 Department of Emergency Medicine, Kusum Dhirajlal (KD) Hospital, Ahmedabad, IND; 3 Department of Medicine, Siddhi Heart and Medical Hospital, Ahmedabad, IND; 4 Department of Medicine, Marengo Care Institute of Medical Sciences (CIMS) Hospital, Ahmedabad, IND

**Keywords:** road travel accident, india, marshall ct score, in-hospital mortality, rotterdam ct score, traumatic brain injury

## Abstract

Objective

Head trauma of any severity, including concussions and skull fractures, can cause a traumatic brain injury (TBI). Prognostication plays a vital role in the scenario of urgency put forth by TBI. The application of CT-based scoring systems developed by the Rotterdam CT score and Marshall classification system appears to be appropriate for the early and precise prediction of clinical outcomes in TBI patients. The present study was designed to determine the predictive value of the Rotterdam CT score and Marshall classification system for in-hospital mortality in patients with TBI.

Methods

All adult patients (≥ 18 years) with acute traumatic brain injury presented over a period from February 2019 to November 2022 were included. Only those patients who had undergone a plain CT scan of the brain during the initial presentation at the emergency department (ED) were considered. Patients who presented with penetrating brain injury as well as those who died on arrival or who died prior to the initial CT scan of the brain were excluded. A total of 127 patients were included in the final data analysis. Based on initial CT-scan findings, the Rotterdam CT score and Marshall classification system were calculated in order to predict in-hospital mortality.

Results

The study was dominated by male patients (85.8%) as compared to female patients (14.2%). The overall mortality rate was 32.3% (n = 41). The mortality rate among males and females was 30.3% (33/109) and 44.4% (8/18), respectively. As per the Glasgow Coma Scale (GCS) classification, the severity of the injury was mild in 12.6% of the study subjects, moderate in 22%, and severe in 65.4%. The mortality rate among the patients with mild severity was 12.5% (2/16), while it was 28.6% in moderate (8/28) and 37.3% (31/83) in the severe category group. The best cut-off point of the Rotterdam score for predicting mortality was >4 (as per the Youden Index), which had a sensitivity and specificity of 60.98% and 90.70%, respectively, while the cut-off point of the Marshall CT classification for predicting mortality was >3 (as per the Youden Index), which had a sensitivity of 82.93% and a specificity of 75.58%. There was only a minor difference in the area under the curve (AUC) value of the receiver operating characteristic curve (ROC) curve between the Rotterdam CT score (0.827) and the Marshall classification system (0.833).

Conclusion

The Rotterdam and Marshall CT scores have demonstrated significant independent prognostic value and may serve as a useful initial evaluation tool for risk stratification of in-hospital mortality among patients with TBI.

## Introduction

Head trauma of any severity, including concussions and skull fractures, can cause traumatic brain injury (TBI), which is frequently referred to as the "silent epidemic". Variable estimates place the annual incidence of TBI worldwide between 27 and 69 million [1]. Low- and middle-income countries (LMICs) bear a disproportionate share of the global burden of TBI-related disability and death. The voluminous burden of TBI cases in India is unknown, but estimates suggest that more than a million trauma-related deaths occur in India annually, with TBIs accounting for half of these deaths [2]. In India, road traffic accidents (RTAs) are by far the leading cause of TBIs, accounting for 60% of all reported cases. Falls and assaults account for an additional 25% and 10% of TBIs, respectively [3].

TBI can cause mild changes in consciousness all the way up to a permanent comatose state and death. In extreme cases, there is widespread damage and swelling throughout the brain [4]. Hence, prognostication plays a vital role in the scenario of urgency put forth by TBI, which not only helps in the determination of the ideal management strategy and judicious allocation of resources but also in predicting the clinical outcome during admission time. The Glasgow Coma Scale (GCS) is a clinically validated method for grading the severity of TBI, typically used to categorize cases as mild, moderate, or severe. However, the reduced inter-rater reliability of GCS scoring, especially the motor component, may reduce its precision in emergency scenarios, making it inaccurate for categorizing severe TBI [5], where immediate triage is of paramount importance.

Computed tomography (CT) and magnetic resonance imaging (MRI) aid in diagnosing TBI. Despite the fact that magnetic resonance imaging is more reliable in diagnosing even mild TBI lesions, it is not always possible to use it in emergency settings due to certain constraints such as accessibility, susceptibility to motion artifacts, and cost [6]. Therefore, the utilization of morphological characterization through CT scans presents a feasible approach for the prompt and impartial assessment of TBI severity in emergency settings. Another added advantage of this modality is its easy availability and rapid image acquisition capabilities [7]. Therefore, the application of CT-based scoring systems developed by Marshall (1995) and Rotterdam CT (2005) appears to be appropriate for the early and precise prediction of clinical outcomes in TBI patients [8]. The three key findings evaluated by the Marshall scoring system are the state of the perimesencephalic cisterns, midline structural deviation, and high- or mixed-density lesions that rely on the volume of the lesion. The Rotterdam score proposed revalued the components of Marshall's classification and added traumatic subarachnoid hemorrhage (tSAH), intraventricular hemorrhage (IVH), and epidural hematomas in order to create an ordinal score criterion [9,10]. The advantages of these prediction models, like reproducibility, minimum interobserver variability, and simplicity of use, make them a promising scoring system [11]. There is a paucity of data in the literature on Indian demographics regarding the utility of these scoring systems in predicting in-hospital mortality in patients with TBI. Hence, the present study was designed to determine the predictive value of the Marshall classification system and Rotterdam CT score for in-hospital mortality in patients with TBI.

## Materials and methods

A retrospective observational study was undertaken on patients with TBI who presented to the emergency department (ED) of Apex Hospital, a tertiary care center in Ahmedabad, Western India. All adult patients (≥ 18 years) with acute traumatic brain injury who presented over a period from February 2019 to November 2022 were included. Only those patients who had undergone a plain CT scan of the brain during the initial presentation at the ED were considered. Patients who presented with penetrating brain injury as well as those who died on arrival or who died prior to the initial CT scan of the brain were excluded.

A standardized abstraction tool was created for the data collection of demographic characteristics that included age, gender, mechanism of injury, type of head injury, GCS score at ED, and all the CT scan findings, including subarachnoid hemorrhage (SAH), epidural hemorrhage (EDH), midline shift (MLS), brain contusion, skull fractures, and subdural hemorrhage (SDH). Based on initial CT-scan findings, the Rotterdam and Marshall CT scores were calculated in order to predict in-hospital mortality. The CT scan reports were assessed by two trained independent radiologists who were blinded to the patient’s conditions, treatment, and final outcome. In the event of disagreements between the two radiologists, the interpretation of the senior radiologist was taken into consideration. The severity of TBI was based on the GCS score at presentation: mild head injury: GCS 13-15; moderate head injury: GCS 9-12; severe head injury: GCS 3-8.

A total of 127 patients were included for final data analysis and were divided into two groups: the survivors' group, which comprised 86 patients who presented to the ED and were subsequently discharged, and the non-survivors' group, which comprised 41 patients who had died during the course of management. The primary outcome measured was in-hospital mortality. A total of four patients were excluded from the data analysis as the deaths had happened due to secondary causes (other than TBI).

For categorical variables, the baseline patient characteristics were presented as frequencies, while for continuous variables, the data were represented as means and standard deviations. Receiver operating characteristic curves (ROCs) were constructed in order to study the discriminatory power of the scoring system. The cut-off point in the prediction of mortality based on the Rotterdam and Marshall CT scores was calculated using the Youden Index. All statistical analysis was performed using MedCalc® Version 20.118 (MedCalc Software Ltd., Ostend, Belgium), and a P-value of <0.05 was considered statistically significant.

This study was carried out in accordance with ethical standards that were later reviewed by the Institutional Review Board (IRB) and deemed exempt.

## Results

In the ROC curve of the Marshall score for predicting mortality with the Marshall CT class > 3, an AUC of 0.833 was obtained, which had a sensitivity of 82.93% (67.9%-92.8%) and a specificity of 75.58% (65.1%-84.2%). The subjects of the study were predominantly male (85.8%) as compared to female (14.2%) (Table 1).

**Table 1:** Demographics, clinical characteristics, and injury severity in patients with TBI RTA: road traffic accident; TBI: trauma brain injury; CT: computed tomography; SDH: subdural hematoma; EDH: epidural hematoma; SAH: subarachnoid hemorrhage

Variable	Total (%)	Survivors (N=86)	Non-survivors (N=41)	P-value
Gender				
Male	109 (85.8%)	76 (88.4%)	33 (80.5%)	0.2336
Female	18 (14.2%)	10 (11.6%)	8 (19.5%)	
Age (years)				
18-39	63 (49.6%)	40 (46.5%)	23 (56.1%)	0.2059
40-59	38 (29.9%)	30 (34.9%)	8 (19.5%)	
≥ 60	26 (20.5%)	16 (18.6%)	10 (24.4%)	
Injury mechanism				
RTA	107 (84.3%)	76 (88.4%)	31 (75.6%)	0.0206
Falls	14 (11%)	5 (5.8%)	9 (22%)	
Others	6 (4.7%)	5 (5.8%)	1 (2.4%)	
Pupils				
Responsive	91 (71.7%)	68 (79.1%)	23 (56.1%)	0.0015
Unilateral unresponsive	26 (20.5%)	10 (11.6%)	16 (39%)	
Bilateral unresponsive	10 (7.9%)	8 (9.3%)	2 (4.9%)	
TBI severity				
Mild	16 (12.6%)	14 (16.3%)	2 (4.9%)	0.1343
Moderate	28 (22%)	20 (23.3%)	8 (19.5%)	
Severe	83 (65.4%)	52 (60.5%)	31 (75.6%)	
CT scan findings				
SDH	36 (28.3%)	21 (24.4%)	15 (36.6%)	0.1548
EDH	19 (15%)	14 (16.3%)	5 (12.2%)	0.5463
SAH	46 (36.2%)	34 (39.5%)	12 (29.3%)	0.2603
Brain contusion	74 (58.3%)	64 (74.4%)	10 (24.4%)	<0.0001
Skull fractures	59 (46.5%)	35 (40.7%)	24 (58.5%)	0.060
Midline shift	21 (16.5%)	11 (12.8%)	10 (24.4%)	0.0999

The majority of the study participants fell into the age range of 18-39 years (49.6%), followed by 40-59 years (29.9%), and then >60 years (20.5%). The most common mechanism of injury was RTA (road traffic accident) (84.3%), followed by falls (11%), and other modes (4.7% that included assaults and other violent injuries). Overall, 71.7% of the patient’s pupils were responsive, and bilateral unresponsive pupils were noticed in 7.9% of the study subjects; 79.1% of the survivors had responsive pupils, 11.6% had unilateral unresponsive pupils, and 9.3% of patients had bilateral unresponsive pupils. For non-survivors, 56.1% of patients had responsive pupils, 39.0% had unilateral unresponsive pupils, and 4.9% of patients had bilateral unresponsive pupils.

As per the GCS classification, the severity of the injury was mild in 12.6% of the study subjects, moderate in 22%, and severe in 65.4% of the patients. The mortality rate among the patients with mild severity was 12.5% (2/16), while it was 28.6% in moderate (8/28) and 37.3% (31/83) in the severe category group. The CT findings of the TBI patients included brain contusions (58.3%), skull fractures (46.5%), subarachnoid hemorrhage (SAH) (36.2%), subdural hematoma (SDH) (28.3%), midline shift (16.5%), and epidural hematoma (EDH) (15%).

The Rotterdam CT score at the time of admission was one in 3.1% of the patients, two in 15.7% of the patients, three in 42.5% of the patients, four in 12.6% of the patients, five in 11.8% of the patients, and a score of six in 14.2% of the patients. The CT findings summarized by the Marshall score at the time of admission were diffusion class I in 3.9% of the patients, class II in 38.6%, class III in 14.2%, class IV in 6.3%, class V in 34.6%, and class VI in 2.4% of the patients, respectively.

The Rotterdam score was significantly lower among the survivors (3.02 ± 1.01) as compared to non-survivors (4.71 ± 1.29; P<0.0001). The same trend was also observed in the Marshall scores of survivors (2.79 ± 1.43) and non-survivors (3.61 ± 1.37; P=0.0027) (Table 2).

**Table 2:** Comparison of the Rotterdam score and the Marshall score between survivors and non-survivors.

Variable	Survivors (N=86)	Non-survivors (N=41)	P-value
Rotterdam score	3.02 ± 1.01	4.71 ± 1.29	<0.0001
Marshall score	2.79 ± 1.43	3.61 ± 1.37	0.0027

In our study, the percentage of mortality based on the Rotterdam CT score with a score of one was 0%, while it was 5% in patients with a score of two, 18.5% for those with a score of three, 31.3% for patients with a score of four, 60% for patients with a score of five, and 88.9% for patients with a score of six. The percentage of mortality in our cohort with the Marshall CT class I was 0%, class II was 6.1%, class III was 22.2%, class IV was 37.5%, class V was 65.9%, and class VI was 66.7% (Table 3).

**Table 3:** The comparison of the Rotterdam and Marshall scoring systems between patients in terms of mortality.

Scoring system	Total (%)	Mortality (%)
Rotterdam CT score		
1	4 (3.1%)	0 (0%)
2	20 (15.7%)	1 (5.0%)
3	54 (42.5%)	10 (18.5%)
4	16 (12.6%)	5 (31.3%)
5	15 (11.8%)	9 (60.0%)
6	18 (14.2%)	16 (88.9%)
Marshall CT class		
Diffusion class I	5 (3.9%)	0 (0%)
Diffusion class II	49 (68.6%)	3 (6.1%)
Diffusion class III	18 (14.2%)	4 (22.2%)
Diffusion class IV	8 (6.3%)	3 (37.5%)
Diffusion class V	44 (34.6%)	29 (65.9%)
Diffusion class VI	3 (2.4%)	2 (66.7%)

The best cut-off point of the Rotterdam score for predicting mortality was >4, which had a sensitivity and specificity of 60.98% and 90.70%, respectively, while the cut-off point of the Marshall CT class for predicting mortality was > 3 (as per the Youden Index), which had a sensitivity of 82.93% and a specificity of 75.58% (Table 4).

**Table 4:** The receiver-operating characteristic (ROC) curve analysis for the Rotterdam score and the Marshall score ROC: receiver operating characteristic; CI: confidence interval

Parameter	Rotterdam CT score	Marshall CT class
Area under the ROC curve (AUROC)	0.827	0.833
95% CI	0.749 to 0.888	0.757 to 0.893
Associated criterion (Youden Index)	>4	>3
Sensitivity (95% CI)	60.98 (44.5 - 75.8)	82.93 (67.9 - 92.8)
Specificity (95%CI)	90.70 (82.5 - 95.9)	75.58 (65.1 - 84.2)

**Figure 1:**
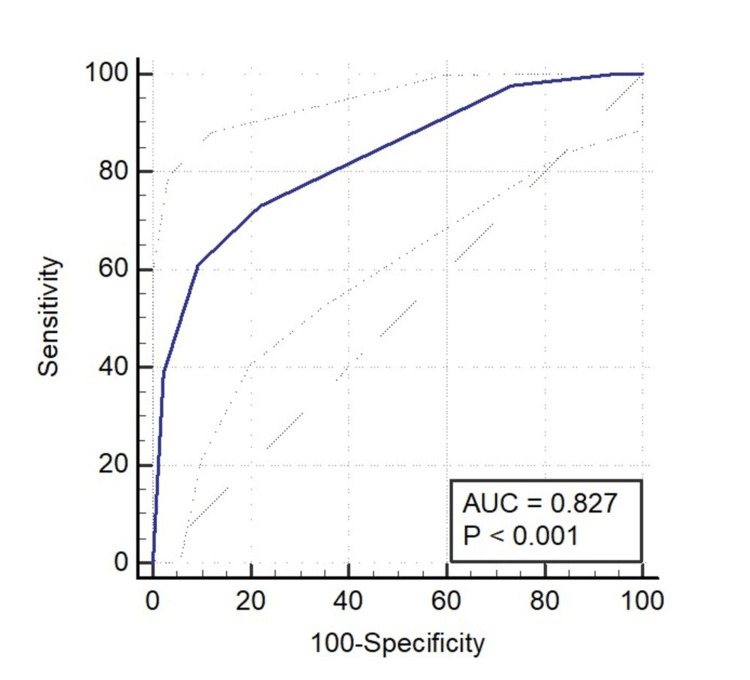
ROC curve of the Rotterdam score for predicting mortality. Considering the Rotterdam CT score > 4, an area under the curve (AUC) of 0.827 was obtained, which had a sensitivity of 60.98% (44.5%–75.8%) and a specificity of 90.70% (82.5%–95.9%).

**Figure 2:**
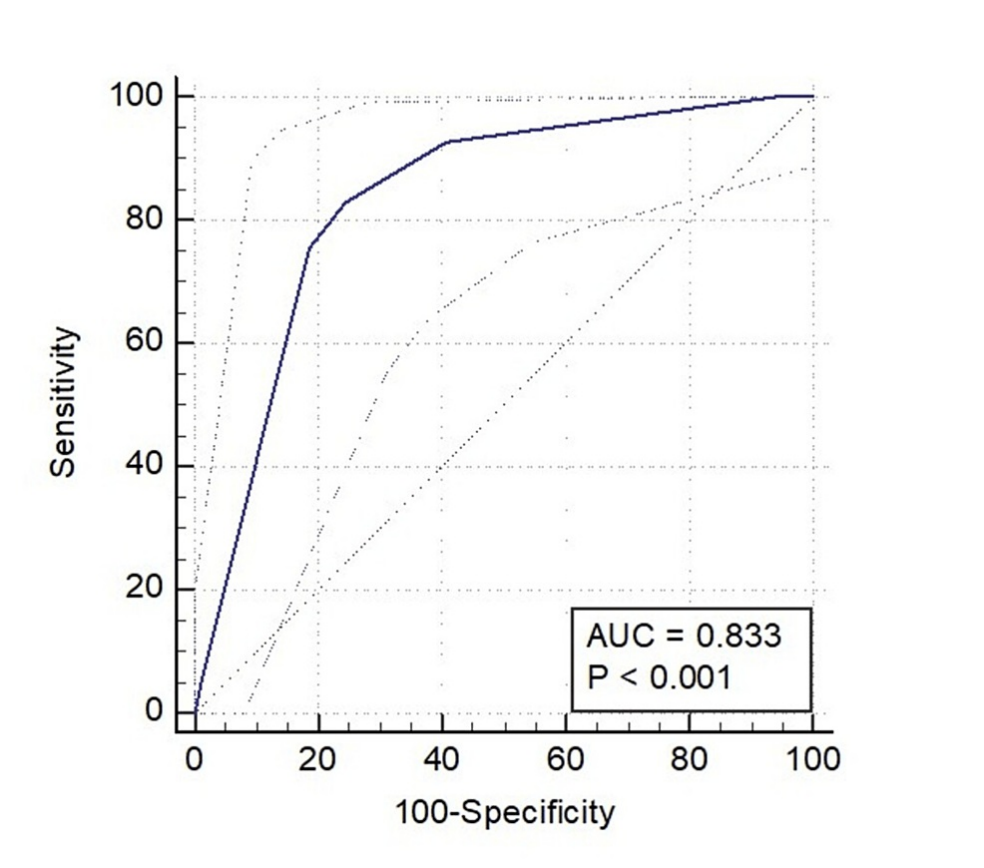
ROC curve of the Marshall score for predicting mortality. With the Marshall CT class > 3, an AUC of 0.833 was obtained, which had a sensitivity of 82.93% (67.9%–92.8%) and a specificity of 75.58% (65.1%–84.2%).

There was only a minor difference in the AUC value of the ROC curve between the Rotterdam CT score (0.827) and the Marshall CT score (0.833) (Figures 1-2).

## Discussion

The present retrospective study analyzed the records of 127 patients with TBI who presented to the ED in order to predict in-hospital mortality using the Marshall classification system and Rotterdam CT scoring systems. The overall mortality rate in the present study was 32.3%. The mortality rate among males and females was 30.3% (33/109) and 44.4% (8/18), respectively. The mortality rate was highest for patients >60 years (38.5%), followed by patients between 18 and 39 years (36.5%) and 40 and 59 years (21.1%). The incidence of brain contusion (74.4%) was most prevalent among the survivors, while skull fractures (58.5%) were more prevalent among the non-survivors. A study by Asim et al. observed that the major head injury lesion observed in survivors and non-survivors was a skull fracture [11].

The overall mortality rate observed in the present cohort was 32.3%, which was higher than Asim et al. (23%) and Bahloul et al. (29.1%) [11,12]. In the present study, the mean Rotterdam score was 3.02 ± 1.01 in survivors, which was lower as compared to 4.71 ± 1.29 in non-survivors, and the difference was found to be statistically significant (P<0.0001). These findings were consistent with those of Asim et al. (2.56 ± 0.94 and 3.78 ± 1.20 for survivors and non-survivors, respectively) and Deepika et al. (2.9 ± 1.2 and 3.9 ± 1.7 for survivors and non-survivors, respectively) [11,13]. The mortality rate increased linearly with an increase in the Rotterdam score, with the lowest rate in patients with a score of one (0%) and the maximum in patients with a score of six (88.9%). A similar linear increase in mortality was also observed in the Marshall CT class, with mortality being lowest in diffusion class I (0%) and highest in diffusion class VI (66.7%). Moreover, there exists a significant difference in the Marshall score between the survivors and non-survivors (2.79 ± 1.43 vs. 3.61 ± 1.37, respectively; P=0.0027) which is similar to Asim et al. and Deepika et al. [11,13].

In our study, the sensitivity and specificity for the Rotterdam score > 4 in predicting in-hospital mortality were 60.98% and 90.70%, respectively, and the values for the Marshall class >3 were 82.93% and 75.58%, respectively. In a study by Asim et al., the Rotterdam scoring system exhibited a sensitivity of 61.2% and a specificity of 85.6% in predicting in-hospital mortality. Additionally, the Marshall scoring system demonstrated a sensitivity of 74.3% and a specificity of 76.3% [11]. In the current study, the ROC analysis demonstrated that both the Rotterdam and Marshall scoring systems accurately predicted in-hospital mortality (Rotterdam score, AUC = 0.827; Marshall score, AUC = 0.833). Our results concur with those of Mata-Mbemba et al. (AUC = 0.85 for both) and Helmy et al. (AUC of the Marshall CT score = 0.848; AUC of the Rotterdam scoring systems = 0.850) [8,14]. In contrast to our findings, studies by Maas et al. and Bobinski et al. observed lower AUCs for Rotterdam (AUC = 0.71 and 0.72) and Marshall scores (AUC = 0.67 and 0.66) [8,15]. Notably, this disparity could be related to the fact that the current study employed in-hospital mortality as the outcome, whereas prior studies investigated the result after a period of follow-up.

As the nature of our study is retrospective and was carried out at a single center, there are some limitations too. In addition to the small sample size, factors between the event and arrival in the ED or acute care were not taken into consideration. Another limitation is that we only considered the CT scan at the time of admission rather than the worst CT scan, which may have better predictive power. However, Yu et al. stated that regardless of the initial brain CT scan time after the trauma, the total Rotterdam score was able to predict mortality and unfavorable outcomes in patients with TBI [16]. Another limitation is the lack of follow-up, as the reason for mortality cannot be ascertained for many of the patients.

## Conclusions

RTA is still one of the leading mechanisms of injury in trauma patients who present in the emergency department. The observed male gender bias towards in-hospital mortality may be attributed to the disproportionate representation of male subjects in the study population. The Rotterdam and Marshall CT scores have demonstrated significant independent prognostic value and may serve as a useful initial evaluation tool for risk stratification of in-hospital mortality among patients with TBI.
